# Assessment of Apple Watch Series 6 pulse oximetry and electrocardiograms in a pediatric population

**DOI:** 10.1371/journal.pdig.0000051

**Published:** 2022-08-22

**Authors:** Lauren Littell, Lisa Roelle, Aarti Dalal, George F. Van Hare, William B. Orr, Nathan Miller, Jennifer N. Avari Silva

**Affiliations:** 1 Department of Pediatrics, St. Louis Children’s Hospital, St. Louis, Missouri, United States of America; 2 Division of Pediatric Cardiology, Department of Pediatrics, Washington University School of Medicine, St. Louis, Missouri, United States of America; 3 Electrophysiology Laboratory, St Louis Children’s Hospital, St. Louis, Missouri, United States of America; 4 Department of Biomedical Engineering, Washington University McKelvey School of Engineering, St. Louis, Missouri, United States of America; McGill University, CANADA

## Abstract

**Background:**

Recent technologic advances have resulted in increased development and utilization of direct-to-consumer cardiac wearable devices with various functionality. This study aimed to assess Apple Watch Series 6 (AW6) pulse oximetry and electrocardiography (ECG) in a cohort of pediatric patients.

**Methods:**

This single-center, prospective study enrolled pediatric patients ≥ 3kg and having an ECG and/or pulse oximetry (SpO2) as part of their planned evaluation. Exclusion criteria: 1) non-English speaking patients and 2) patients in state custody. Simultaneous tracings were obtained for SpO2 and ECG with concurrent standard pulse oximeter and 12-lead ECG. AW6 automated rhythm interpretations were compared to physician over-read and categorized as accurate, accurate with missed findings, inconclusive (automated interpretation: “inconclusive”), or inaccurate.

**Results:**

A total of 84 patients were enrolled over a 5-week period. 68 patients (81%) were placed into the SpO2 and ECG arm, with 16 patients (19%) placed into the SpO2 only arm. Pulse oximetry data was successfully collected in 71/84 (85%) patients and ECG data in 61/68 (90%). ΔSpO2 between modalities was 2.0±2.6% (r = 0.76). ΔRR was 43±44msec (r = 0.96), ΔPR 19±23msec (r = 0.79), ΔQRS 12±13msec (r = 0.78), and ΔQT 20±19msec (r = 0.9). The AW6 automated rhythm analysis yielded a 75% specificity and found: 1) 40/61 (65.6%) “accurate”, 2) 6/61 (9.8%) “accurate with missed findings”, 3) 14/61 (23%) “inconclusive”, and 4) 1/61 (1.6%) incorrect.

**Conclusion:**

The AW6 can accurately measure oxygen saturation when compared to hospital pulse oximeters in pediatric patients and provide good quality single lead ECGs that allow for accurate measurement of RR, PR, QRS, and QT intervals with manual interpretation. The AW6-automated rhythm interpretation algorithm has limitations for smaller pediatric patients and patients with abnormal ECGs.

## Author summary

Wearable health devices are becoming more commonly available to the general public. These devices are often being used without doctor prescription or guidance so there is increasing need to understand how to interpret and use the data they provide. The Apple Watch Series 4 and newer have the capability to produce an on-the-spot single lead electrocardiogram (ECG) that is intended to detect atrial fibrillation in adult patients. Additionally, the Apple Watch Series 6 (AW6) has a function to get on-the-spot and background measurements of blood oxygen saturation which has not been studied yet. This study aimed to study the AW6 ECG and pulse oximetry functions in pediatric patients with and without heart disease. We found that the AW6 produces good quality single-lead ECGs that can be accurately interpreted by pediatric cardiologists, but the watch’s automated rhythm interpretation is not reliable in children, particularly those with abnormal ECGs. The AW6 pulse oximetry was accurate when compared to standard hospital pulse oximetry, but did produce some notable outliers, so this data must be used with caution and verified by medical grade pulse oximetry. This data paves the way for further studies and use of the AW6 in medical care of pediatric patients.

## Introduction

Cardiac wearable digital health devices have become common in the general population with some devices requiring physician prescription, and others being marketed direct to consumer [1]. This unprecedented access to data raises questions of regulation, testing, and perhaps most importantly, validity of the data. Each of these devices have specific indications for use and targeted use populations. Devices that can be purchased over the counter will undoubtedly have wide reaching consequence beyond the intended use. Cardiac electrophysiology remains at the forefront of these technologies, with much of the testing and algorithm development focusing on adult patients with atrial fibrillation.

Children represent an important population to consider for the use of these devices, with 42% of young children owning their own tablet device [2] and 53% of children owning a smart phone by age 11 (a number that increases to 84% by teenage years) [3]. In the pediatric community, there has been an increase in use of wrist-worn wearable devices, including Apple Watch and Fitbit, though the prevalence has not been rigorously quantified. Given the increased usage of these technologies, there has been an appropriate increase in questions of how to interpret these data in pediatric patients.

The Apple Watch Series 4 and subsequent generations added the ability to obtain on-demand recording of a single lead electrocardiogram (ECG). It has been studied in adults and has received FDA clearance to detect atrial fibrillation in adults 22 years or older [4]. Apple Watch Series 6 (AW6) is distinct from previous versions in that it also functions as a pulse oximeter to estimate blood oxygen saturation on-demand [5]. During an oxygen saturation estimation, the watch emits red and green LED and infrared light, while photodiodes measure the amount of reflected light [5].

The aim of this study is to assess the feasibility and accuracy of the pulse oximetry estimation, ECG algorithm auto-interpretation, and ECG data from the AW6 as compared to clinical standard of care in a diverse cohort of pediatric cardiology patients.

## Methods

After obtaining approval from the Institutional Review Board (IRB) at Washington University in St. Louis, a prospective study was undertaken. Informed written consent was obtained from a parent/guardian and assent was obtained from pediatric patients ≥ 8 years of age when developmentally appropriate. Patients admitted at St. Louis Children’s Hospital or scheduled for outpatient visits in a Pediatric Cardiology Clinic were recruited into one of two study arms: 1) ECG and pulse oximetry or 2) pulse oximetry only. Inclusion criteria were as follows: 1) age < 23 years, 2) weight ≥ 3kg, and 3) ECG ordered as part of their medical care (for ECG and pulse oximetry arm). Exclusion criteria included: 1) non-English speaking patients and 2) patients in state custody. Patients were enrolled into one of four groups based on weight given the importance of surface area contact to the back of the device: Group A) 3-10kg, Group B) >10-18kg, Group C) >18-32kg, and Group D) >32kg. Patient sex, date of birth, height, weight, and cardiac history were obtained through chart review.

Measurements of pulse oximetry and ECG tracings were collected using the AW6, standard pulse oximetry machine (Coviden Nellcor Portable SpO2 Patient Monitoring System, Medtronic Inc, Dublin, Ireland) and 12-lead ECG (GE MAC VU360, GE Healthcare, Wauwatosa, WI). All AW6 ECGs were obtained using version 1 of the Apple ECG application. Pulse oximetry data sets from AW6 and standard pulse oximetry were obtained simultaneously. ECG tracings with the AW6 and standard 12-lead ECGs were obtained simultaneously (when possible) or consecutively (within 2 minutes of each other). The AW6 requires 30-seconds of continuous information to collect an ECG tracing. ECGs were defined as consecutive if there was an overlap in timing between studies.

### Obtaining data from AW6

To obtain a single lead ECG tracing with the AW6 utilizing the ECG application, the watch is placed on the wrist (left wrist for lead I) and a finger from the right hand is held on the watch crown for 30 seconds. To obtain an oxygen saturation measurement, the watch is placed on the skin (typically wrist) and the watch directs the user to remain still for 15 seconds. A 15 second countdown appears on the watch until a numerical result or “unsuccessful measurement” is displayed.

Younger patients, who may lack coordination, had their finger lightly held in place by a parent or research team member taking care that only the patient’s finger touched the AW6 crown. For smaller patients in Group A, the small surface area of their finger often led to failure to obtain a full 30-second tracing prompting the watch to begin the 30-second counter over again. The data collection protocol could then be modified to place the base of infants’ palm on the watch crown in lieu of a single finger. Similarly, modifications specific to Group A when obtaining AW6 pulse oximetry data included placing the watch on areas with more surface area (i.e., palm of hand, dorsum of foot, or around calf/thigh) which at times allowed for measurements to be obtained when measurement on the wrist had previously failed.

Paper copies of AW6 tracings and 12-lead ECGs were de-identified, stripped of automated interpretations, and batch interpreted by two board-certified pediatric electrophysiologists. Heart rhythm and ECG intervals (RR, PR, QRS, and absolute QT) were documented on each data set. Physician over-read of AW6 tracings was compared to 12-lead and to automated interpretation from AW6 (ECG application version 1). For each ECG tracing, alignment between the AW6 algorithm interpretation and the physician over-read was classified as follows: 1) accurate (true negative or true positive; AW6 algorithm interpretation consistent with physician over-read), 2) accurate with missed findings (true negative or true positive regarding arrhythmia; AW6 algorithm interpretation consistent with physician over-read of rhythm, but additional abnormal ECG finding(s) present), 3) inconclusive (AW6 algorithm result was “inconclusive” when tracing over-read was normal sinus rhythm or sinus with additional abnormal findings), or 4) incorrect (false positive; AW6 algorithm reported atrial fibrillation when tracing was over-read as normal sinus rhythm).

### Statistical analysis

Summary data are presented as frequency with percentage. Demographic data are presented as means with range. Bland Altman analyses and intra-class correlation (SPSS, IBM, Chicago, IL) were used to compare modalities.

## Results

### Demographic aata

Demographic data for the overall cohort and subgroups are presented in **Table 1**. Structural heart disease was present in 34/84 (41%) patients, electrical abnormalities in 12/84 (14%), and a combination of structural heart disease and electrical abnormalities in 7/84 (8%). There was no cardiac history in 31/84 (37%) patients.

**Table 1:** Demographic data. Patient demographic data is shown. Diagnoses in this table were part of the patients’ known history before study data was obtained. Structural diagnoses included: Coarctation of the aorta, cardiomyopathy, single ventricle physiology, multisystem inflammatory syndrome in children (MIS-C) and coronary artery anomaly, orthotopic heart transplant (OHT), pulmonary hypertension, rhabdomyoma, tetralogy of Fallot (TOF), ventricular septal defect (VSD), atrial septal defect (ASD), patent ductus arteriosus (PDA), or atrioventricular (AV) canal defect. Electrical diagnoses included: Supraventricular arrhythmias, AV block, catecholamine polymorphic ventricular tachycardia (CPVT), long QT syndrome, or premature atrial/ventricular conductions. Concomitant structural and electrical diagnoses included: Double outlet right ventricle and SVT, MIS-C + coronary artery anomaly and AV block, OHT and atrial flutter, TOF s/p pacemaker, cardiomyopathy s/p pacemaker, congenitally corrected transposition of the great arteries s/p pacemaker, and TOF and Wolff-Parkinson-White.

Patient Characteristics	All Groups (n = 84)	Group A (n = 19)	Group B (n = 18)	Group C (n = 14)	Group D (n = 33)
Sex Female Male	46 (55%) 38 (45%)	12 (63%) 7 (37%)	7 (39%) 11 (61%)	8 (57%) 6 (43%)	19 (58%) 14 (42%
Age (yrs) Average (range)	7.2 (0.1–18)	0.4 (0.1–1)	2.2 (1.2–4.2)	6.9 (4.5–10.7)	14 (8–18)
Weight (kg) Average (range)	31.5 (3.8–167.2)	5.9 (3.8–8.9)	13.3 (10.2–16.8)	24.5 (18.1–31.8)	59.6 (34.2–167.2)
Height (cm) Average (range)	114.8 (52–189.7)	60.8 (52–70)	87.4 (77.5–100)	117.8 (88–139.5)	159.5 (129–189.7)
Cardiac Diagnosis(es)					
None	31 (36.9%)	3 (15.8%)	4 (22.2%)	4 (28.6%)	20 (60.6%)
Structural	34 (40.5%)	15 (78.9%)	9 (50%)	5 (35.7%)	5 (15.2%)
Electrical	12 (14.3%)	1 (5.3%)	4 (22.2%)	4 (28.6%)	3 (9.1%)
Concomitant Structural and Electrical	7 (8.3%)	0 (0%)	1 (5.6%)	1 (7.1%)	5 (15.2%)

**Fig 1:**
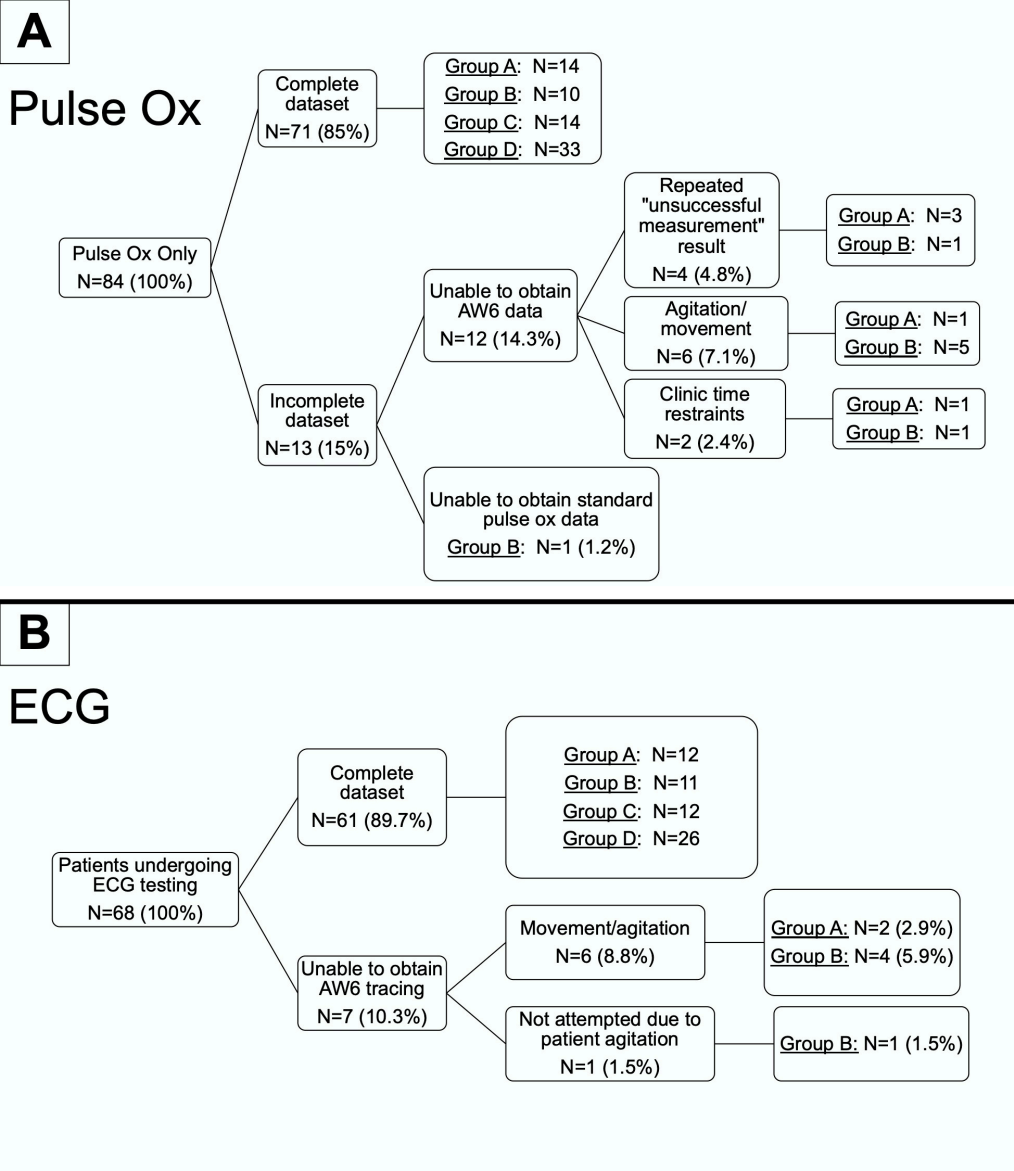
Patient flowchart for pulse oximetry **(A)** and ECG **(B)** data. Abbreviations: AW6 = Apple Watch 6. Group A = 3-10kg, Group B = >10-18kg, Group C = >18-32kg, and Group D = >32kg.

A total of 84 patients were recruited with 68 (81%) entering the pulse oximetry and ECG arm and 16 (19%) entering the pulse oximetry only arm. In patients who had ECG data, a total of 61/68 (90%) completed both Apple Watch 6 and standard 12-lead ECGs. Paired ECG data sets were not available for 7 (10%) patients due to an inability to complete the full 30 second AW6 tracing due to movement and/or agitation. All 7 patients with incomplete ECG data were from either groups A (n = 2, 14.3%) or B (n = 5, 31.3%). (See **Fig 1A**)

Complete paired sets of pulse oximetry data were collected in 71/84 (85%) patients. Pulse oximetry data was incomplete in 13/84 (15%) of patients, with 12/13 patients not having AW6 data and 1/13 not having standard pulse oximetry data. Inability to obtain AW6 pulse oximetry data was due to repeated “unsuccessful measurement” results, which were related to patient agitation/movement or clinic time restraints. All patients with incomplete AW6 pulse oximetry data were from either groups A (n = 5) or B (n = 7), resulting in 26.3% of Group A and 38.9% of Group B with no AW6 oxygen saturation to compare to standard (see **Fig 1B**).

**Fig 2:**
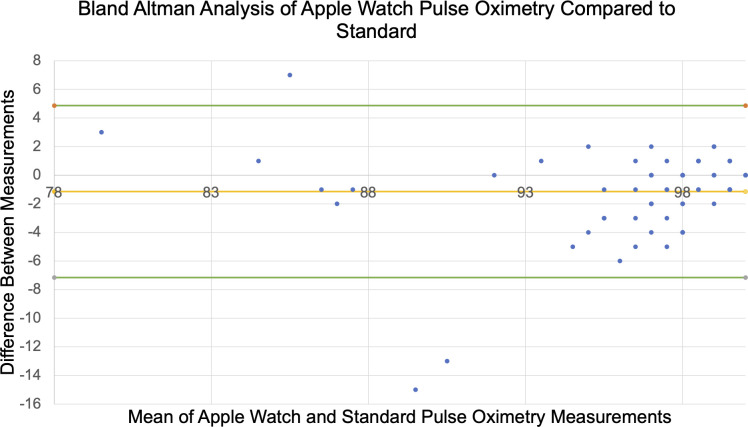
Bland Altman Analysis of AW6 pulse oximetry compared to standard. Horizontal lines represent the mean difference of oxygen saturation between the Apple Watch 6 and standard pulse oximetry and the upper and lower limits of agreement (LOA). Upper and lower LOA represent +1.96 and -1.96 standard deviations respectively. Points may represent multiple patients.

*Pulse Oximetry Data*: Average absolute difference between AW6 and standard pulse oximetry oxygen saturation measurements was 2.0±2.6 (r = 0.76) (See **Table 2**). Most patients (n = 65/71, 91.5%) had oxygen saturations ≥90% on standard pulse oximetry (See **Fig 2**). Six patients (8.5%) had hypoxemia <90% SpO2 on standard pulse oximetry with an average absolute difference between the AW6 and standard oxygen saturation measurements of 2.5±2.3. There were 4/71 (5%) saturation measurements that differed by >5% SpO2 (differences of 6, 7, 13, and 15 points), with 3 from group D and 1 from Group A. All three of the measurements from Group D were normal saturations (>95%) on hospital measurement and the watch underestimated by 6, 13, and 15 (watch read 93, 84, and 82 respectively). After adjustment of the watch face to adjust and maximize skin contact, these 3 patients underwent repeat assessment resulting in an average difference of 2.3±1.5. The patient from Group A with standard and AW6 measurements with difference >5% was a hypoxemic patient with SpO2 82% on hospital measurement and the watch reported 89%, overestimating by 7.

**Table 2:** Difference between AW6 oxygen saturation and ECG intervals compared to hospital pulse oximeter and 12-lead ECG. SD = standard.

Absolute difference between AW6 and SD	All Groups (n = 61)	Group A (n = 12)	Group B (n = 11)	Group C (n = 12)	Group D (n = 26)	Correlation (r)
Oxygen Saturation	2.0 ± 2.6	2.3 ± 1.9	1.4 ± 1.4	1.5 ± 1.6	2.1 ± 3.4	0.76
RR (msec)	42.5 ± 44.2	40.4 ± 52.4	44.1 ± 33.8	30.0 ± 24.5	48.7 ± 51.3	0.96
PR (msec)	19.3 ± 22.7	15.4 ± 8.4	18.2 ± 14.2	22.1 ± 14.5	28.1 ± 47.3	0.79
QRS (msec)	12.4 ± 13.3	5.8 ± 5.1	8.6 ± 8.7	14.2 ± 15.5	16.2 ± 15.3	0.78
QT absolute (msec)	20.2 ± 19.1	19.2 ± 18.7	16.4 ± 9.5	28.8 ± 26.5	18.5 ± 18.3	0.90

### ECG interval data

**Fig 3:**
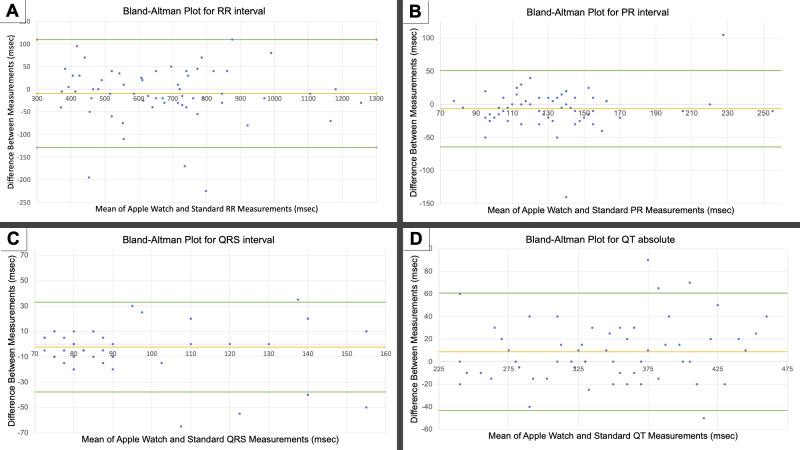
Bland Altman Analysis of AW6 ECG interval measurements compared to standard ECG: **(A**) RR, **(B)** PR, **(C)** QRS, **(D)** QT absolute. Horizontal lines represent the mean difference of oxygen saturation between the Apple Watch 6 and standard pulse oximetry and the upper and lower limits of agreement (LOA). Upper and lower LOA represent +1.96 and -1.96 standard deviations respectively. Points may represent multiple patients.

**Fig 4:**
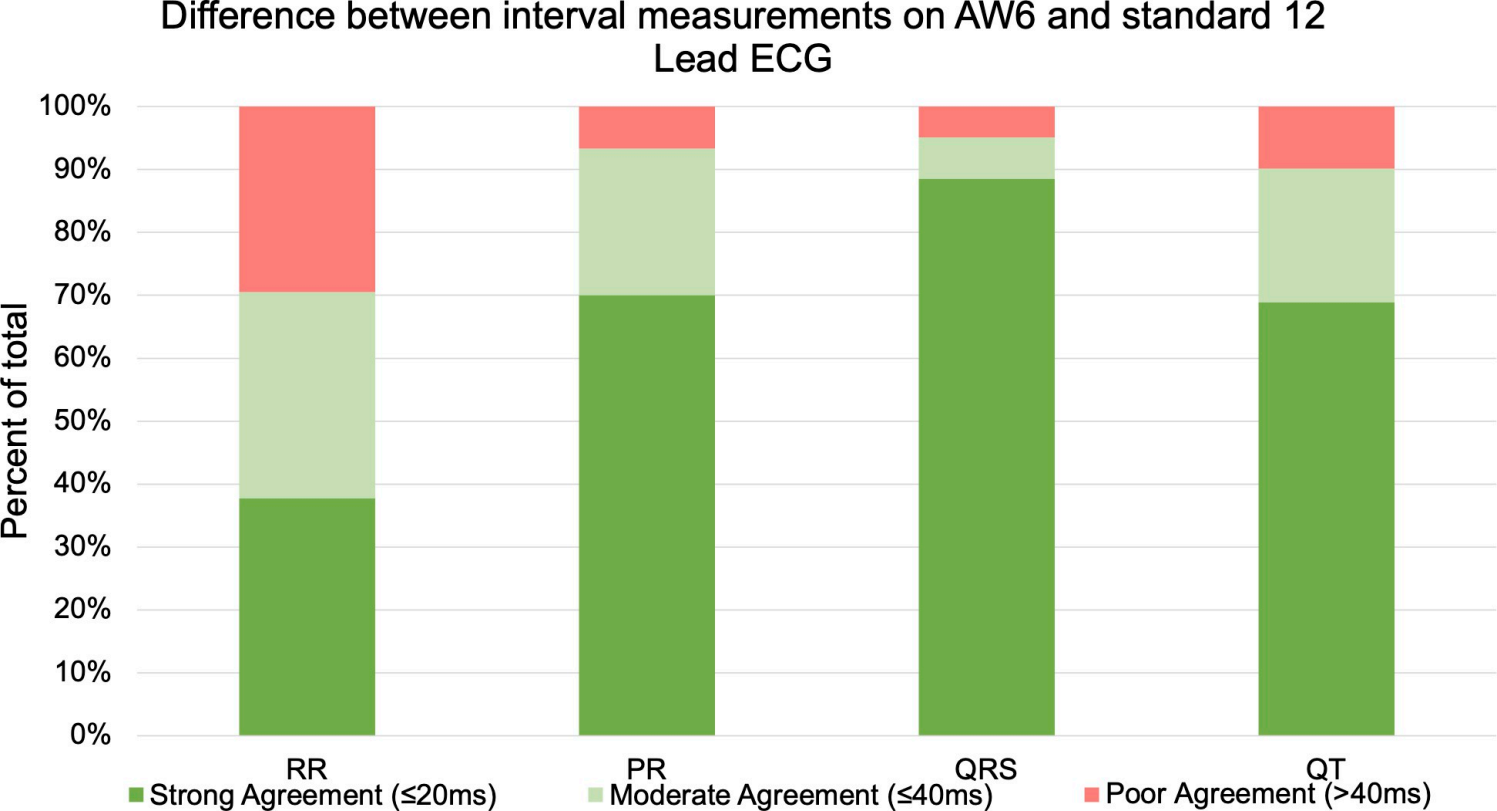
Level of agreement in different ECG intervals between AW6 and 12-lead ECG.

When comparing all enrolled patients’ Apple Watch 6 and standard 12-lead ECG interval measurements, the ΔRR was 42.5±44.2 msec (r = 0.96), ΔPR was 19.3±22.7 msec (r = 0.79), ΔQRS was 12.4±13.3 msec (r = 0.78), and ΔQT absolute was 20.2±19.1 msec (r = 0.90) (**See Table 2 and Fig 3**). Moderate agreement (≤40msec difference) was present in 212/243 total intervals (87.2%): 43/61 RR (70.5%); 56/60 PR (93.3%); 58/61 QRS (95.1%); QT absolute 55/61 (90.2%). Perfect agreement (≤20msec difference) was present in 161/243 total intervals (66.3%): 23/61 RR (37.7%); 42/60 PR (70%; PR was unable to be measured in one set of tracings due to presence of pacing artifact); 54/61 QRS (88.5%); QT absolute 42/61 (68.9%) (See **Fig 4**).

### ECG algorithm

**Fig 5:**
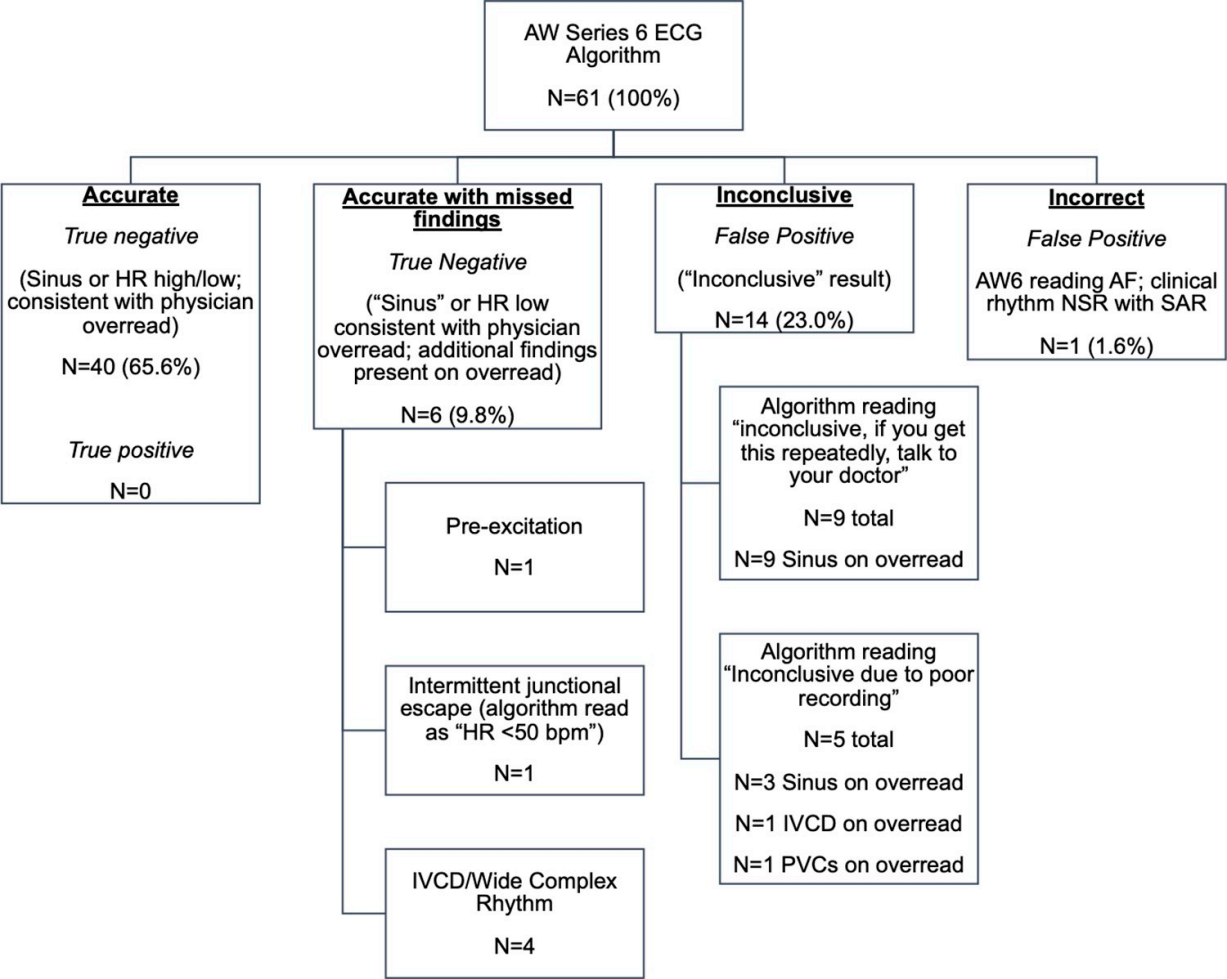
Analysis of AW6 automated Interpretation algorithm. Of note, no patients were in an acute arrhythmia at the time of ECG collection. Abbreviations: AF = atrial fibrillation, HR = heart rate, IVCD = intraventricular conduction delay, NSR = normal sinus rhythm, PVCs = premature ventricular conductions, SAR = sinus arrhythmia.

Manual overread of rhythm on AW6 tracings and 12-lead ECGs displayed 100% agreement between modalities. Categorization of the AW6 automated rhythm analysis compared to physician over-read into 4 groups found: 1) 40/61 (65.6%) to be “accurate” (all true negatives), 2) 6/61 (9.8%) to be “accurate with missed findings” (all true negatives), 3) 14/61 (23%) to be “inconclusive” (false positive as it may induce anxiety in the patient/parent and lead to the patient seeking medical care or testing), and 4) 1/61 to be incorrect (false positive regarding arrhythmia) (See **Fig 5**). This resulted in a specificity of 75% for the algorithm, with sensitivity not calculated given there were no true positive patients.

**Fig 6:**
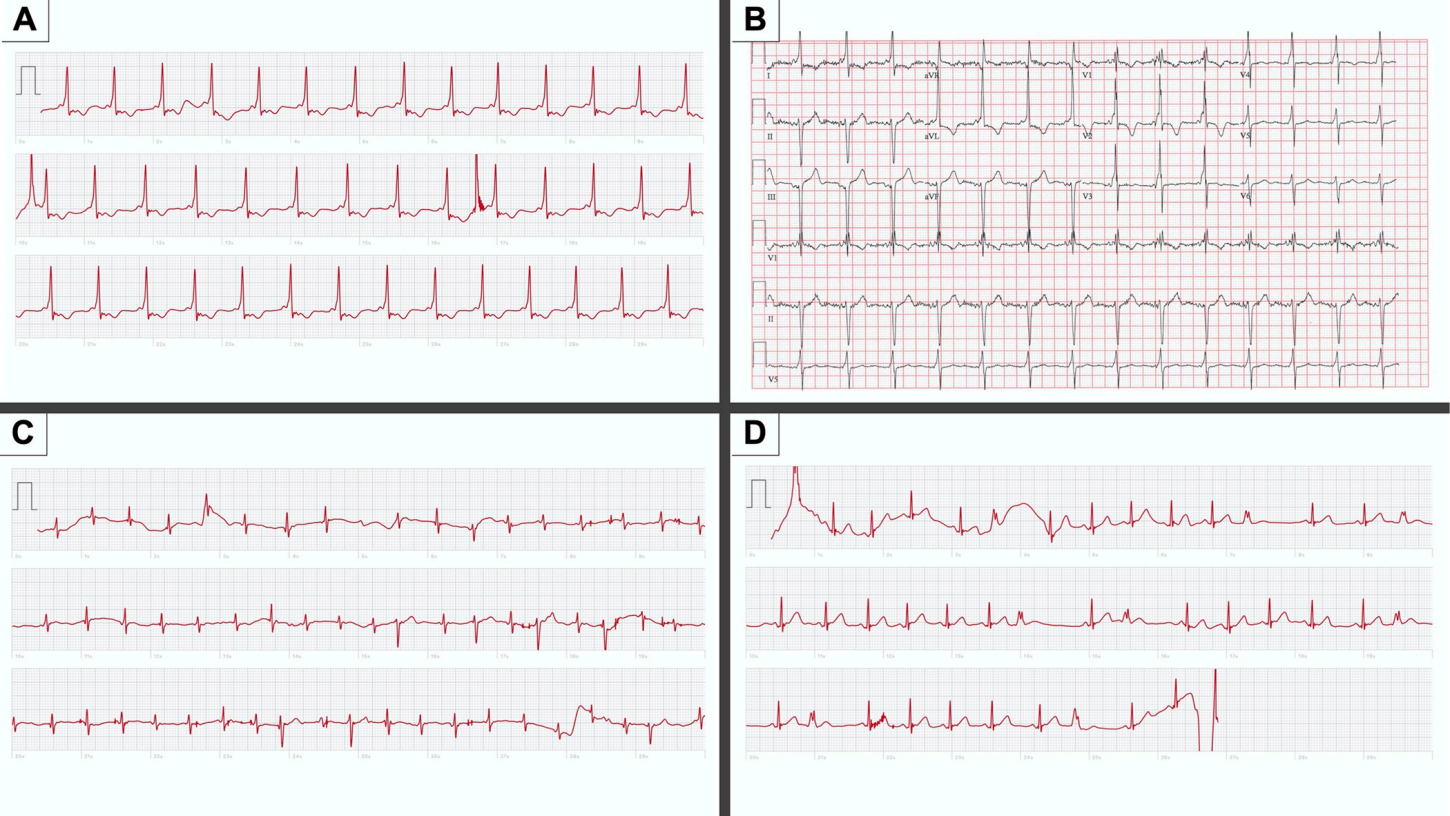
Apple Watch 6 ECG examples. Simultaneously obtained AW6 and 12-lead ECGs of a patient with WPW. Pre-excitation is evident on both the **(A)** AW6 tracing and **(B)** 12-lead ECG. **(C)** AW6 tracing of a 6-year-old female. The AW6 automated interpretation incorrectly labeled this tracing as atrial fibrillation. Physician over-read was sinus rhythm with PACs. **(D)** AW6 tracing of a 2-year-old female capturing frequent PVCs. The AW6 automated reading was “inconclusive due to poor recording). This tracing also demonstrates how movement artifact appears on AW6 tracings. Additionally, this was the only time a tracing less than 30 seconds saved during the study. Typically, if contact was broken before 30 seconds, the tracing would not save and the 30 second process needed to be started over.

Further analysis of the “accurate with missed findings” group was notable for 6 tracings with AW6 readings of sinus rhythm or HR less than 50, but with additional clinically relevant abnormal findings on physician over-read. Clinically relevant findings noted in this group by physician over-read included: 1/6 with bradycardia with intermittent junctional escape (AW6 interpretation was “heart rate less than 50”), 1/6 with ventricular pre-excitation (AW6 interpretation of sinus rhythm), and 4/6 with IVCD or wide complex rhythm (AW6 interpretation of sinus rhythm). The “inconclusive” group was notable for 9/14 (64%) with AW6 readings of “inconclusive, if you get this repeatedly, talk to your doctor” with all 9 being over-read by physicians as normal sinus rhythm. The other 5/14 (36%) had AW6 readings of “inconclusive due to poor recording” with 3 over-read as normal sinus rhythm, 1 as sinus rhythm with interventricular conduction delay (IVCD), and one as sinus rhythm with PVCs (See **Fig 6** for example AW6 tracings that show WPW, PACs–incorrectly called atrial fibrillation by watch, and PVCs).

## Discussion

Our study assesses the use of the AW6 pulse oximetry and ECG in a heterogeneous pediatric cardiology population with 63% of patients having a known cardiac disease/history. The data show that the AW6 can accurately measure oxygen saturation when compared to hospital pulse oximeters in pediatric patients and provide good quality single lead ECGs that allow for accurate measurement of RR, PR, QRS, and QT intervals with manual interpretation. However, the AW6 ECG interpretation algorithm (ECG version 1) did not demonstrate strong accuracy and has notable limitations in pediatric patients.

Currently, the gold standard method of assessing accuracy of pulse oximeters is to compare measurements to arterial blood samples with normal oxygen saturations and at varying levels of hypoxemia [6,7]. The AW6 did not undergo this type of comparative testing to gain FDA clearance and is instead market direct to consumers for non-medical use. In this study, we demonstrated good correlation and agreement between the AW6 and a standard hospital pulse oximetry machine. These results function as an early feasibility and usability study but are insufficient to validate the AW6 pulse oximeter for medical use.

Portable and wearable devices capturing on-demand ECGs have significant potential in outpatient monitoring of pediatric patients, particularly those with a known arrhythmia history [8,9,10]. However, few studies have been done to validate ECGs produced by wearable devices in pediatric patients. Gropler et al [11] studied the Kardia device in pediatric patients with and without a known cardiac history and found that it produced accurate ECGs when comparing manual over-read of rhythm and interval measurements by two pediatric electrophysiologists to consecutively obtained 12-lead ECGs. Similar studies have been done with the Apple Watch 4 in neonates and healthy adults [12,13]. Recently, Kobel et al [14] published their work focused on the utility of the Apple Watch series 4 ECG (iECG) in pediatric patients (ages 0–16 years) and showed excellent correlation (K>0.7, p<0.01) in all ECG interval measurements between the AW ECG and standard 12 lead ECG with reliable AW ECG rhythm interpretation in 95% of cases.

Our study found moderate agreement (≤40ms difference) was present in >90% of all PR, QRS, and QT absolute intervals which is similar to what was demonstrated in Saghir’s AW study in healthy adults and Kobel’s study in pediatric patients [13,14]. Perfect/strong agreement (≤20ms difference) was most often present in PR and QRS intervals (70 and 89% respectively) and less common in RR and QT absolute intervals (38 and 69% respectively). The decreased incidence of perfect agreement in RR and QT intervals is likely multifactorial, including increased beat-to-beat variation coupled with a larger scale of error with longer interval measurements.

Additionally, the data presented here show more difficulty in obtaining AW6 pulse oximetry and ECG tracings in infants and toddlers due to patient movement and cooperation. This finding was not recapitulated in the analysis by Kobel et al [14] as there was no age- or weight-based subgroup analysis performed. In our study, we found that the use of alternate sites for obtaining data (the palm of hand, dorsum of foot, or around calf/thigh) provided alternate sites for data collection. This difficult in data acquisition, though surmountable, may potentially limit the usability of the AW6 in the youngest and smallest patient populations.

In addition to the potential for cardiologists to utilize AW6 ECGs for outpatient monitoring of intervals, this study aimed to test the reliability of the AW ECG automated rhythm interpretation. The AW6 ECG algorithm for detection of atrial fibrillation has previously been validated in adults. This study further reinforced the poor accuracy of the AW automated rhythm interpretation in pediatric patients demonstrated by Kobel et al [14], which is markedly different than the perfect agreement previously demonstrated in healthy adults (Saghir et al [13]). The algorithm primarily designed to interpret adult ECGs and our study population including a high number of abnormal baseline ECGs, increased baseline artifact due to movement, and the wide range of normal heart rates in infants and pediatric patients. Additionally, the AW rhythm algorithm (version 1) is limited to certain outputs, such as sinus rhythm, high/low heart rate, or atrial fibrillation. In our population, the algorithm failed to recognize sinus rhythm in some patients providing an “inconclusive” output, and in one patient had a false positive result for atrial fibrillation. Ideally, future versions of the algorithm would include ability to determine other abnormalities such as IVCD, ectopy, and pre-excitation.

The ability to accurately manually interpret AW6 ECGs has the potential to be a valuable tool for outpatient monitoring, potentially including real-time data collection during telehealth visits, the potential to act as an event monitor for palpitations, and monitoring and management of QT prolonging medications. The specificity of the AW6 v1 ECG algorithm was 75%, which is markedly lower than reported in the adult data [13] demonstrating the importance of ECG interpretation in the context of age. Compared to other recognized medical screening tests such as mammography (specificity 90% [15]), Papanicolaou testing (specificity 86–100% [16]) or prostate screening antigen testing (specificity 94% [17]), the ECG algorithm for AW6 v1 underperformed.

### Study limitations

This was a single center study at an academic University hospital involving patients presenting to Cardiology clinic or admitted to the inpatient cardiology service which underrepresents healthy pediatric patients compared to the general population. Significant limitations in the comparison of the AW6 pulse oximetry to standard hospital pulse oximeters are present as there are not well-defined methods of direct comparison of two pulse oximeters. There was a large portion of ECGs in this study (23%) that needed to be obtained consecutively (within 2 minutes of each other), rather than simultaneously, which may contribute to differences in interval measurements when comparing modalities. Varying amounts of baseline waveform interference was present on the 12-lead ECGs when the AW6 tracings were obtained simultaneously, which may have decreased precision of measurements. Further variability in measurements could have stemmed from the difference in time each device records with standard 12-leads recording 10 seconds and the AW6 producing a 30 second tracing. Pediatric patients also have a high rate of heart rate variability and sinus arrhythmia which likely contributes particularly to variability in RR and QT measurements.

## Conclusion

The AW6 can be used to obtain pulse oximetry and good quality single lead ECGs in a broad pediatric population. The automated ECG interpretation should not be used for clinical decision making or screening given the relatively low specificity, but manual interval and rhythm interpretation by cardiologists may be beneficial to outpatient monitoring of pediatric cardiology patients.
